# IL-6 Deficiency Attenuates Murine Diet-Induced Non-Alcoholic Steatohepatitis

**DOI:** 10.1371/journal.pone.0007929

**Published:** 2009-11-20

**Authors:** Emmanuel Mas, Marie Danjoux, Virginie Garcia, Stéphane Carpentier, Bruno Ségui, Thierry Levade

**Affiliations:** 1 INSERM, U858, Toulouse, France; 2 Institut de Médecine Moléculaire de Rangueil, Université Toulouse III Paul-Sabatier, Equipe n°14, IFR31, Toulouse, France; 3 Unité de Gastroentérologie, Hépatologie et Nutrition, Département de Pédiatrie, Hôpital des Enfants, Toulouse, France; 4 Département d'Anatomopathologie, Hôpital Purpan, Toulouse, France; 5 Laboratoire de Biochimie Métabolique, Institut Fédératif de Biologie, Hôpital Purpan, Toulouse, France; The University of Hong Kong, Hong Kong

## Abstract

**Background:**

The role of inflammation in the pathogenesis of non-alcoholic steatohepatitis (NASH), a common cause of liver disease, is still poorly understood. This study aimed at assessing the involvement of a major inflammatory cytokine, IL-6, in NASH.

**Materials and Methods:**

Steatohepatitis was induced by feeding wild-type or IL-6^−/−^ mice for 5 weeks with a methionine and choline-deficient (MCD) diet.

**Results:**

Whereas MCD diet-induced weight loss and decreases in serum glucose, cholesterol and triglyceride levels were similar in both genotypes, serum alanine aminotransferase was less elevated in IL-6^−/−^ mice than in wild-type animals. Despite having a comparable liver steatosis score, IL-6-deficient mice exhibited less lobular inflammation than their wild-type littermates. Liver gene expression of TGF-β and MCP-1 was also strongly attenuated in mutant mice; a more modest reduction was observed for PPAR-γ and F4/80 transcripts as well as proteins. Chromatographic analysis of liver lipids demonstrated that MCD diet induced in normal and mutant mice a similar decrease in the ratio of phosphatidylcholine to phosphatidylethanolamine. However, the diet-induced increase in the levels of sphingomyelin and ceramide was less important in IL-6^−/−^ mice.

**Conclusion:**

Altogether, these results indicate that IL-6 deficiency does not block the development of NASH; yet, IL-6 plays a critical role in the accompanying liver inflammation.

## Introduction

The term non-alcoholic steatohepatitis (NASH) was first used in 1980 by Ludwig et al. [1] to describe a histological pattern that mimicked alcoholic hepatitis but occurred in patients without history of alcohol abuse. The features of NASH on liver biopsy include steatosis, inflammation, liver cell injury and varying degrees of fibrosis. NASH belongs to the spectrum of non-alcoholic fatty liver disease (NAFLD) and is becoming a major public health problem because it is associated with obesity, insulin resistance and the metabolic syndrome. Therefore, NASH is believed to affect approximately 3% of adults in Western countries and represents, together with alcohol and hepatitis C virus infection, one of the main etiologies of cirrhosis [2].

A ‘two-hit process’ has been proposed to underlie the pathophysiology of NASH [3]. According to this concept, in the first hit, there is an increase of circulating free fatty acids resulting in liver steatosis. This step is enhanced by insulin resistance, which appears to play a prominent role. Secondary insults (‘the second hit’) include oxidative stress, whereby production of radical oxygen species and lipid peroxidation occur, recruitment of inflammatory cells and dysregulated cytokine/adipokine production. This induces hepatocyte cell death, by apoptosis or necrosis, and subsequent liver inflammation and fibrosis. A current, more integrated hypothesis suggests the involvement of multiple and interconnected events [4].

Whereas the molecular mechanisms leading from liver steatosis to NASH (or from NASH to cirrhosis) still remain unclear, hepatic inflammatory cell recruitment appears as a key step, and the contribution of inflammatory cytokines such as tumor necrosis factor (TNF)-α or interleukin-6 (IL-6) seems obvious. Nevertheless, despite recent work on TNFα in the pathogenesis of NASH, the role of this pro-inflammatory cytokine is still a matter of debate. TNFα is known to play a central role in insulin resistance [5] and is critically involved in alcoholic steatohepatitis [6]. Moreover, liver and adipose tissue TNFα and TNF receptor 1 (TNFR1) transcripts [7] as well as serum TNFα levels [8] are increased in patients with NASH. While these observations point to some contribution of TNFα to the pathogenesis of NASH, recent studies on animal models have led conflicting conclusions. For instance, deficiency of TNF receptors did not prevent elevation of serum ALT in *ob/ob* mice [9] or after intragastric overfeeding of a high-fat diet [10]. However, TNFR1-deficient mice fed a high sucrose diet did not manifest steatosis [11], and liver steatosis and fibrosis were attenuated in doubly TNFR1/TNFR2-deficient mice fed a methionine and choline-deficient (MCD) diet [12]. With regard to IL-6, a pleiotropic cytokine which regulates inflammatory responses and represents another putative mediator of steatohepatitis, its precise role in NASH is almost unknown (see Discussion).

The present study was therefore undertaken to clarify the place of IL-6 in the development of NASH. For this purpose, IL-6-deficient (IL6^-/-^) mice were analysed. We used MCD diet to induce experimental NASH because of the reproducibility of this well-established model that allows the assessment of the inflammatory pathway despite the absence of insulin-resistance [13]–[15]. This is a frequently employed nutritional model, where steatosis appears and serum ALT levels increase after 3 weeks, followed by focal inflammation and fibrosis after 5 and 8 weeks, respectively. In this model, lipid storage is believed to be the consequences of increased fatty acid uptake and decreased VLDL secretion [16], [17]. The histological changes are morphologically similar to those observed in human NASH. Our biochemical, histological and molecular analyses indicate that in mice IL-6 contributes to the MCD diet-induced liver inflammation.

## Materials and Methods

### Ethics Statement

All animal experimentation was conducted in accordance with accepted standards of humane animal care (recommendations of the European Accreditation of Laboratory Animal Care). Mouse experiments were approved and performed according to the guidelines of the Toulouse University Midi-Pyrénées Regional Animal Safety Committee.

### Animal Experiments

Ten-week-old C57BL/6 male mice, either WT or deficient for IL-6, were fed a MCD diet (MP Biomedicals, France) or a normal diet (2016 Teklad Global 16% Protein Rodent Diet) for 5 weeks. IL-6^−/−^ mice [18] were kindly provided by Dr. M. Thomsen (Inserm U858, Toulouse, France). Animals had unrestricted access to food and water, were housed in temperature-controlled rooms (in the specific pathogen-free animal facility of IFR31, Toulouse) and kept on a 12-hour light/dark cycle. Mice were anaesthetised with fluothane inhalation and about 300 µl of blood was collected by retro-orbital puncture. Mice were sacrificed by cervical dislocation. Livers were rapidly excised and rinsed in phosphate buffered saline (PBS). Two slides of the largest lobe were fixed in 10% formaldehyde for histological analyses and the remaining liver was immediately frozen at −80°C for other analyses.

### Morphologic Studies

Liver was embedded into paraffin and sections were cut and stained with hematoxylin eosin (HE), Oil Red O or Masson trichrome. Evaluation was performed by a single blinded pathologist (M.D). The criteria used were steatosis, lobular and portal inflammation, portal fibrosis, cellular ballooning, lipogranuloma, and apoptosis, according to Brunt et al. [19]. Based on these criteria, the NAFL (non-alcoholic fatty liver) Activity Score was calculated, which allows to distinguish NASH (NAFL score≥5), borderline (3 to 4) or no NASH (<3) [20].

Immunohistochemistry of paraffin-embedded liver sections was carried out using an anti-F4/80 antibody (MCA497, Serotec) to characterize macrophagic cells.

### Blood Tests

Serum was collected after a 20 min centrifugation at 1000 x g. Glucose levels were measured using a Glucotrend 2® analyser (Roche Diagnostics, France), and triglyceride, cholesterol and ALT levels were determined using a Cobas MIRA analyser.

### Liver Lipid Analyses

A 100 mg portion of the liver was transferred into 1 ml of buffer (10 mM Hepes-KOH pH 7.4, 42 mM KCl, 5 mM MgCl_2_, 1 mM dithiothreitol, 0.5% CHAPS, 1 µM PMSF, and 2 µg/ml leupeptin). Liver was homogenised at 4°C using a Turrax homogeniser (2×10 sec), followed by sonication (2×5 sec) with a tip Soniprep sonicator. An aliquot of liver homogenate was centrifuged for 10 min at 500 x g at 4°C. The supernatant was used for protein analysis by the Bradford method [21] and stored for further analyses. Lipids were extracted according to Folch et al [22]. Briefly, 300 µl of distilled water and 2.5 ml of chloroform/methanol (2/1, by vol.) were added to 200 µl of liver homogenate; after vigorous mixing and centrifugation, the lower organic phase was isolated and evaporated under a nitrogen stream.

Total liver phospholipids were determined by measuring the content of inorganic phosphorus [23]. For quantification of sphingomyelin, the lipid extract was first submitted to mild alkaline methanolysis (to degrade acylglycerophospholipids) and resistant lipids (i.e., sphingomyelin) were reisolated for determination of the phosphorus content as described above. Individual glycerophospholipid species were separated by high performance liquid chromatography (Lipidomic core, IFR30, Toulouse). Ceramide levels were determined by using *E. coli* diacylglycerol kinase and [γ^32^P]-ATP as previously described [24].

### Liver Lipid Peroxide Determination

Total lipoperoxides were measured as thiobarbituric acid-reactive substances (TBARS) in 50 and 100 µl of liver homogenate [25]. Briefly, after addition of water (to a final volume of 1 ml), 0.5N HCl (100 µl) and thiobarbituric acid (1 ml), and heating at 95°C in the darkness for 20 min, butanol (2 ml) was added. After centrifugation for 10 min at 500 g at 4°C, the fluorescence intensity of the supernatant was recorded using a Jobin-Yvon spectrofluorimeter (at 515 nm and 548 nm for the excitation and emission wavelength, respectively). TBARS were quantified using malondialdehyde (MDA) as a standard.

### Assessment of Apoptosis

Several techniques to assess apoptosis were employed. DEVDase activity was measured by fluorometry on a 100 µl aliquot of the liver homogenate supernatant [26]. Caspase-3 processing was also analysed by Western blotting. Total liver proteins (30 µg) were separated on 15% PAGE. We used a rabbit primary anti-caspase-3 antibody (Cell Signaling); complexes were revealed with a SuperSignal® West Dura Substrate kit (Pierce Biotechnology, USA). Finally, DNA fragmentation was studied. After an overnight digestion of 100 mg of liver with proteinase K at 55°C, DNA was extracted using a chloroform/phenol method. Twenty-five µg of DNA were migrated on 2% agarose gel containing ethidium bromide.

### Determination of mRNA Levels

RNA extraction was performed using RNA STAT-60® (Ams Biotechnology, UK). cDNA was obtained using 1 µg of total RNA for reverse transcription and the Superscript II® Reverse Transcriptase (Invitrogen). Real-time quantitative PCR was carried out using primers for F4/80, GAPDH, HPRT, IL-6, MCP-1, PPAR-γ_1/2_, TGF-β, and TNFα, and with Power SYBR® Green PCR Master Mix (Applied Biosystems, UK). We used an ABI 7900 analyser (Applied Biosystems). Sequences for primers are indicated in Table 1.

**Table 1:** Sequences of primers used for quantitative RT-PCR.

Gene	Sense	Antisense
PPAR-γ_1/2_	CCGAAGAACCATCCGATTGA	TTTGTGGATCCGGCAGTTAAG
TGF-β	GAGCCCGAAGCGGACTACTA	CACTGCTTCCCGAATGTCTGA
MCP-1	GCAGTTAACGCCCCACTCA	CCAGCCTACTCATTGGGATCA
F4/80	TGACAACCAGACGGCTTGTG	GCAGGCGAGGAAAAGATAGTGT
HPRT	TGGCCATCTGCCTAGTAAAGC	GGACGCAGCAACTGACATTTC
GAPDH	TGCACCACCAACTGCTTAGC	GGCATGGACTGTGGTCATGAG

All sequences are oriented, from left to right, 5′ to 3′.

### Western Blotting Analyses

Equal amounts of proteins (50 µg) were extracted from livers, electrophoresed on a 10% SDS-polyacrylamide gel, transferred to a nitrocellulose membrane (Perkin-Elmer) and blotted with anti-Foxp3 (14-7979, eBioscience) and PPAR-γ (sc-7273, Santa-Cruz). Proteins were detected using an ECL detection system (Thermo Scientific). Secondary antibodies were from Cell Signaling Technology.

### Data Analysis

Numerical data are expressed as means±SEM. Statistical analysis was performed using unpaired Student *t* test and Chi^2^ test, for continuous or categorical variables respectively. Values of p<0.05 were considered significant.

## Results

### Effects of MCD Diet on Anthropometric Parameters, Serum Glucose and Lipid Levels

**Figure 1:**
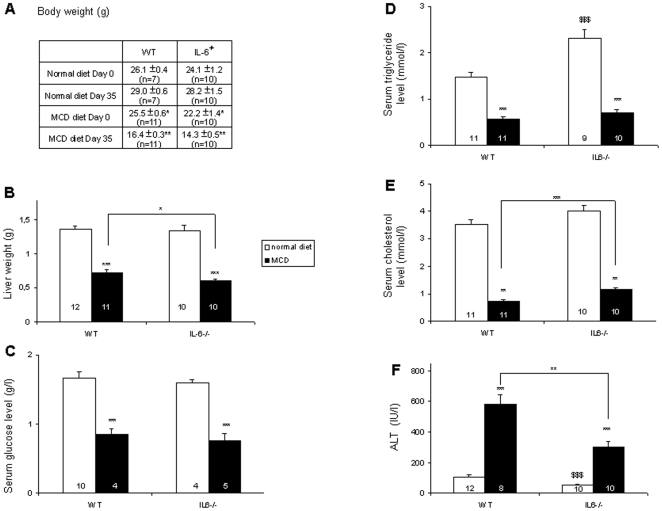
MCD diet results in loss of body and liver weights, hypoglycemia, hypotriglyceridemia, hypocholesterolemia, and increased serum alanine aminotransferase (ALT) activity. After 5 weeks of a MCD diet, body (A) and liver (B) weights, serum levels of glucose (C), triacylglycerols (D), cholesterol (E), and ALT (F) of WT and IL-6^−/−^ mice were determined. Data are means±SEM; the number of animals is indicated into each column. Asterisks denote significant differences (* p<0.05, ** p<0.01, *** p<0.001) between mice of different genotypes on the MCD diet (when indicated above the line) or between different diets for the same genotype (when indicated in A and above the columns). $$$ indicates a difference (p<0.001) between IL-6^−/−^ and WT mice on a normal diet.

WT mice were slightly heavier than IL-6^−/−^ mice but both control and mutant mice lost about one third of their initial body weight after 5 weeks of MCD diet (Fig. 1A). There was no liver enlargement and the liver weight even decreased by about 50% on MCD diet (Fig. 1B). Without fasting, MCD diet induced a marked decrease in serum glucose, triglyceride and cholesterol levels as compared to a normal diet (Fig. 1C, D and E). This decrease was comparable in WT and IL-6^−/−^ mice. Cholesterol levels, however, remained slightly higher in mutant mice.

### Hepatic Injury on MCD Diet

Feeding mice with MCD diet resulted in a 5.5-fold increased level of ALT. This biochemical liver injury induced by MCD diet was similar in WT and IL-6^−/−^ mice (Fig. 1F). Increased ALT levels were already present at 3 weeks (not shown).

**Figure 2:**
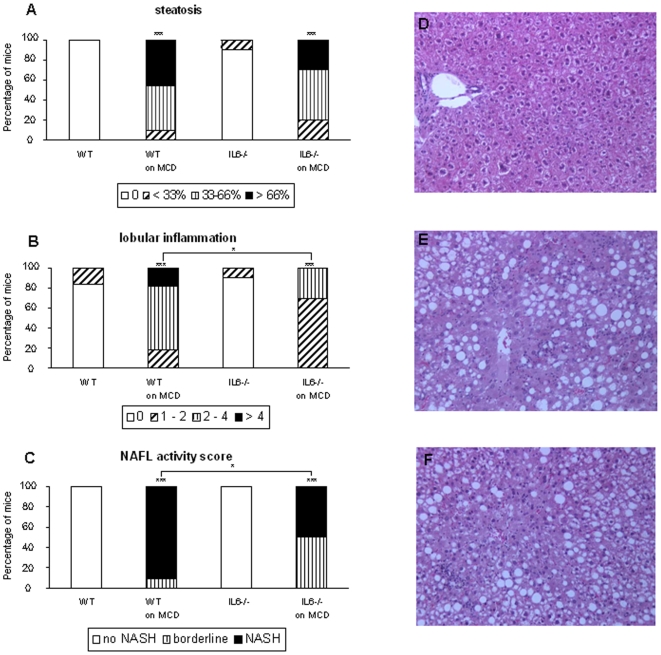
Effect of IL-6 deficiency on MCD diet-induced steatohepatitis. After 5 weeks of a MCD diet, mice were sacrificed and the liver was examined for scoring steatosis (A), ballooning (not shown) and lobular inflammation (B). The NAFL (non-alcoholic fatty liver) activity score was calculated (C). Representative sections of hematoxylin/eosin-stained liver of WT mice on normal (D) or MCD diet (E), or of IL-6^−/−^ mice on MCD diet (F), are shown. Liver sections of WT mice (E) revealed large fat droplets and the presence of inflammatory cells. In IL-6^−/−^ mice, steatosis was similarly present, but the number of inflammatory cells was decreased. The number of animals per group was 12 and 11 for WT mice, and 10 and 10 for IL-6^−/−^mice fed a normal or MCD diet, respectively. * p<0.05, *** p<0.001.

Histological analysis revealed that the steatosis induced by MCD diet was similar for all animals (Fig. 2A). Steatosis was assessed using HE staining but also with Oil Red O (not shown). Cellular ballooning appeared quite mild and comparable for all types of mice (not shown). As illustrated in representative sections, the liver of WT mice fed the normal diet was normal (Fig. 2D) whereas on the MCD diet it contained large fat droplets and clusters of inflammatory cells (Fig. 2E). But the number of inflammatory cells and clusters were reduced in IL-6^−/−^ mice (Fig. 2F). Indeed, the lobular inflammation detected in mice fed the MCD diet was mild to moderate for IL-6^−/−^ mice, whereas it was moderate to severe for WT animals (Fig. 2B). Finally, after MCD feeding, the NAFL Activity Score [20] was lower in IL-6^−/−^mice (p = 0.04) than in WT animals, with respectively 50% and 90% of confirmed NASH (Fig. 2C). After 5 weeks on MCD diet, fibrosis could not be detected on Masson trichrome-stained sections for both genotypes (not shown). Of note, under a normal diet, the steatosis and lobular inflammation scores were similar in WT and IL6-deficient mice.

### Absence of Apoptosis Associated with Steatohepatitis

Development of steatohepatitis, including NASH, has been shown to be associated with liver apoptosis [12], [27]–[32]. In the liver from mice fed the MCD diet for 5 weeks, we did not find any sign of apoptosis whatever the genotype and the NAFL Activity Score. Indeed, there were no increase of DEVDase (effector caspase) activity, no proteolytic cleavage of caspase-3, no DNA fragmentation, and no apoptotic bodies on histological examination. We therefore concluded that, by that time of MCD diet feeding, liver apoptosis is not a prominent phenomenon contributing to NASH.

### Alterations in Liver Lipid Metabolism

**Figure 3:**
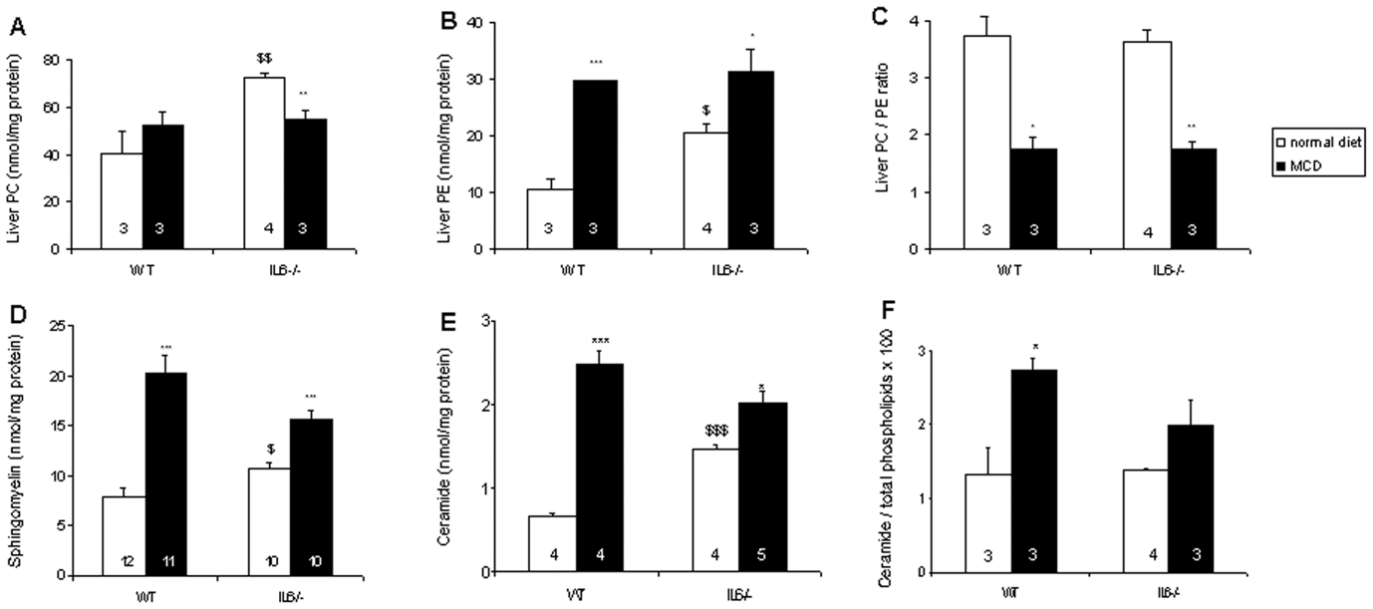
Effects of IL-6 deficiency on liver lipid content. Lipids were extracted from the livers of mice fed a normal or the MCD diet for 5 weeks. Phosphatidylcholine (PC, panel A) and phosphatidylethanolamine (PE, panel B) levels were measured by HPLC, and the PC/PE ratio (C) was calculated. The levels of sphingomyelin (D) were determined by inorganic phosphorus analysis preceded of alkaline methanolysis, and those of ceramide (E) were measured by the diacylglycerol kinase assay. In panel F, ceramide is expressed as the ratio to total phospholipids. Data are expressed as in the legend to Fig. 1. Further symbols ($ p<0.05, $$ p<0.01, $$$ p<0.001) indicate differences between IL-6^−/−^ and WT mice on a normal diet.

Besides the storage of triacylglycerols, steatohepatitis is known to be associated with alterations in liver phospholipid metabolism [33], [34]. Insulin resistance is also associated with increased intracellular content of fatty acids and their metabolites, including ceramide [35], [36]. In addition, cytokines such as TNFα and IL-6, which might participate in the pathogenesis of NASH, elicit changes in sphingolipid metabolism and stimulate the production of bioactive lipids, such as ceramide, which behave as second messengers [36]–[38]. We thus analysed the glycerophospholipid and sphingolipid composition of the livers from mice fed a MCD diet. As shown in Fig. 3C, feeding for 5 weeks the MCD diet resulted in a decrease of the phosphatidylcholine to phosphatidylethanolamine (PC/PE) ratio of approximately 50%. This decrease was comparable for control and IL-6-deficient mice, although the PC levels slightly increased in WT but decreased in mutant mice, in which the basal levels were higher (Fig. 3A). Under conditions of feeding a normal diet, the liver content of total phospholipids (not shown) as well as that of sphingomyelin, the major sphingophospholipid, was higher in IL-6^−/−^ mice than in WT animals (Fig. 3D). The same applied for the ceramide content (Fig. 3E). After 5 weeks of MCD diet, livers exhibited an increase both in the sphingomyelin and ceramide concentrations for all mice. However, whereas the content of these two lipids increased by more than 2.6-fold in WT mice (p<0.001 for sphingomyelin and ceramide), it did not increase by more than 1.5-fold in IL-6^−/−^ animals (p<0.001 for sphingomyelin and p = 0.01 for ceramide). A lesser elevation of ceramide in mutant mice was also observed when normalizing to total phospholipids (Fig. 3F).

### Lipid Peroxidation

**Figure 4:**
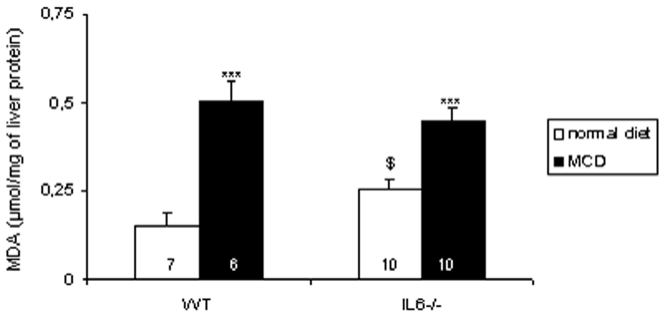
Effect of IL-6 deficiency on MCD diet-induced peroxidation of liver lipids. Total lipoperoxides were measured as TBARS. Each determination was performed in duplicate. Data are means±SEM; the number of animals is indicated into each column. The asterisks denote a significant difference between diets for the same genotype (***, p<0.001), and $ a difference (p<0.05) between IL-6^−/−^ and WT mice on a normal diet.

Because NASH is often associated with lipid peroxidation [25], [31], [32], [39], [40] we next sought to assess the importance of this phenomenon in IL-6^−/−^ mice. Liver peroxides were measured through the quantification of TBARS. Figure 4 shows that, on a normal diet the liver TBARS content was higher (p = 0.03) for IL-6^−/−^ mice than for WT mice. As expected, these levels increased on MCD diet, but this increase was only 1.7-fold in mutant mice (p<0.001) as compared to 3.3-fold in WT mice (p<0.001).

### Reduced Expression of Genes of the Inflammatory and Fibrosing Response in IL-6^−/−^ Mice

**Figure 5:**
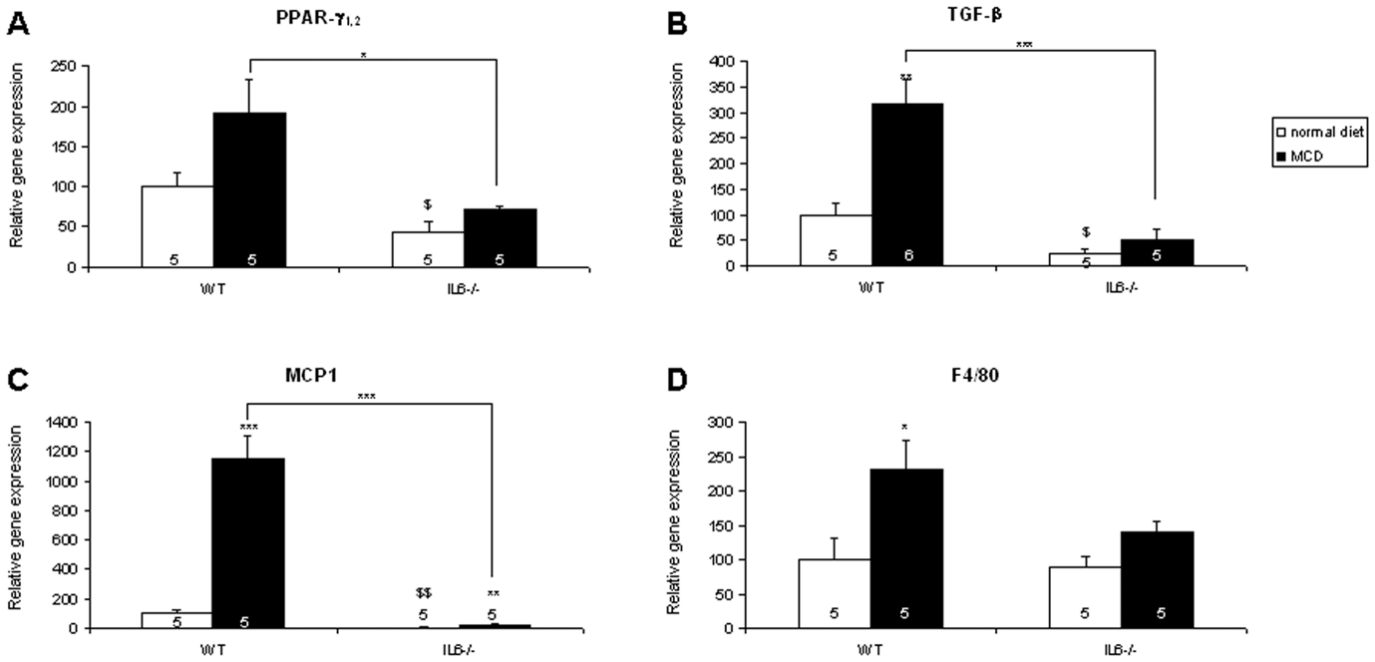
Effects of IL-6 deficiency on MCD diet-induced changes in liver gene expression. The mRNA levels of PPAR-γ_1, 2_ (A), TGF-β (B), MCP-1 (C), and F4/80 (D) were measured by quantitative RT-PCR and normalized to the level of HPRT mRNA. Results are expressed as the percentage of the levels found in WT mice fed a normal diet. Each determination was performed in duplicate. Data are means±SEM; the number of animals is indicated into each column. Asterisks denote significant differences (* p<0.05, ** p<0.01, *** p<0.001) between mice of different genotypes on the same diet (when indicated above the line) or between different diets for the same genotype (when indicated above the columns). Further symbols ($ p<0.05, $$ p<0.01) indicate differences between IL-6^−/−^ and WT mice on a normal diet.

In accordance with the fact that steatosis is associated with hepatic expression of PPAR-γ, MCD diet induced an increased PPAR-γ_1/2_ gene expression in all mice (Fig. 5A). However, the level of PPAR-γ_1, 2_ transcripts was higher in WT mice than in IL-6^−/−^ mice, although a similar difference existed on a normal diet. As illustrated in Fig. 5B, mRNA levels of the fibrogenesis mediator TGF-β significantly increased on MCD diet only in WT mice. The expression level of this gene was lower on MCD diet in IL-6^−/−^ mice than in WT mice. The expression of the monocyte chemoattractant protein MCP-1 increased 11-fold on MCD diet in WT mice (Fig. 5C). Of interest, despite a modest (25–30%) increase on MCD diet, MCP-1 mRNA levels remained extremely low in IL-6^−/−^ mice. Finally, in accordance with the decreased inflammation observed histologically, the mRNA levels of the macrophage marker F4/80 that were similar on a normal diet increased by 233 and 159% on MCD diet in WT and IL-6^−/−^ mice, respectively (Fig. 5D). Although TNF was generally expressed at a lower level than the other genes examined, on MCD diet its levels were increased by 12-fold in WT mice but only 6-fold in IL-6-deficient mice (not shown). While undetectable in mutant mice, the level of IL-6 gene expression in WT animals was not increased after 5 weeks of MCD diet feeding (not shown). This finding would be in agreement with a recent study in which hepatic IL-6 levels were determined after 6 weeks feeding a liquid MCD diet [41] and is likely related to the fact that it was measured at a too late point.

**Figure 6:**
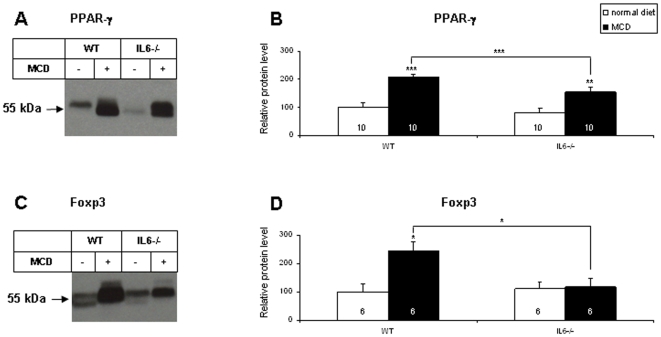
Effects of IL-6 deficiency on MCD diet-induced changes in liver expression of PPAR-γ and Foxp3. Equal amounts of protein (50 µg) were analyzed by Western blotting (representative images on the left panels). Densitometric analysis of the blots is presented on the right panels. Data are means±SEM; the number of animals is indicated into each column. Asterisks denote significant differences (* p<0.05, ** p<0.01, *** p<0.001) between mice of different genotypes on the same diet (when indicated above the line) or between different diets for the same genotype (when indicated above the columns).

To test whether some of the changes observed above for mRNA translated into changes of the corresponding proteins, we performed Western blotting experiments on liver lysates. As illustrated in Fig. 6, the levels of PPAR-γ increased in both genotypes after MCD diet feeding. However, they remained significantly lower in IL-6^−/−^ mice. We also tested the expression of Foxp3, a master transcription factor for TGF-β-induced differentiation of T regulatory cells. Figure 6 shows that induction of Foxp3 during the MCD diet was abolished in IL-6-deficient animals.

**Figure 7:**
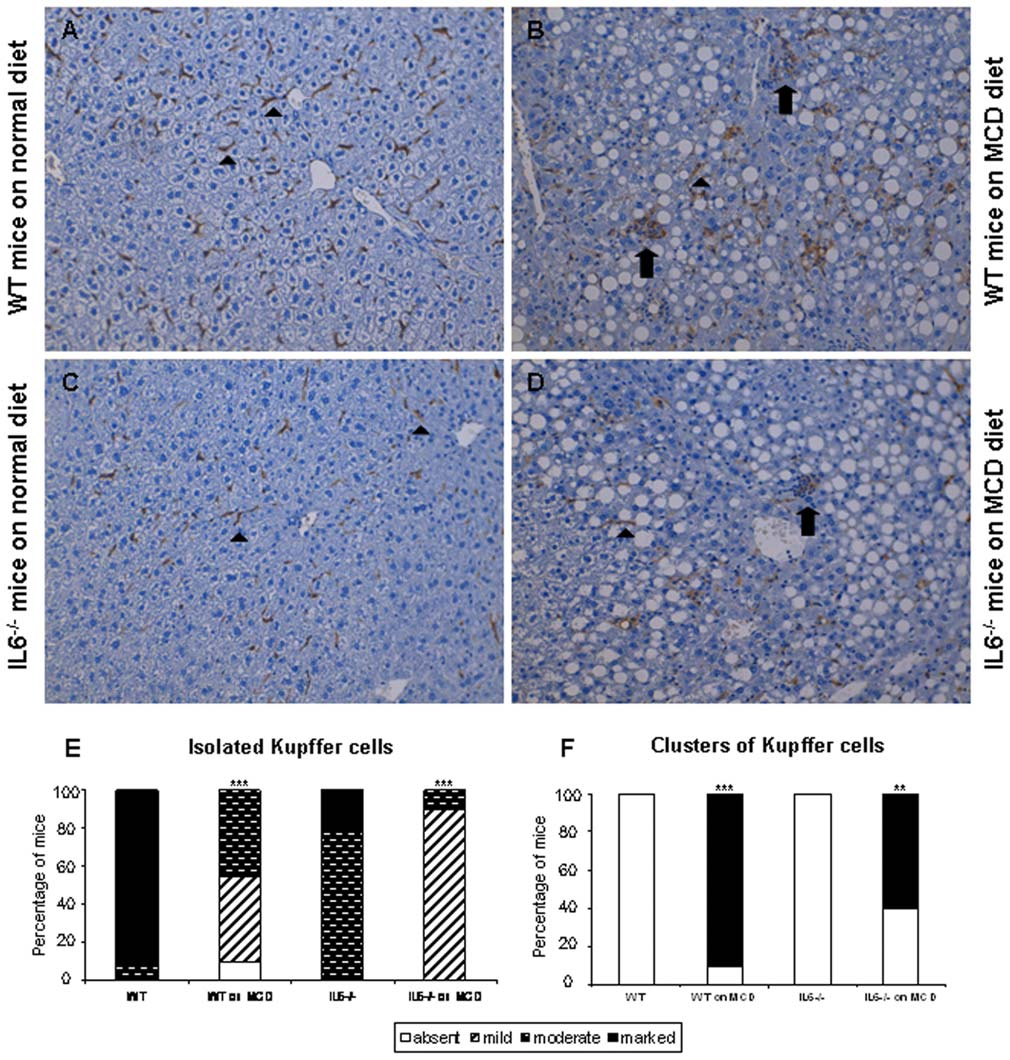
Effect of IL-6 deficiency on Kupffer cell activation during MCD diet-induced steatohepatitis. Representative pictures of immunohistochemical detection of F4/80-positive cells in the liver of WT and IL-6^−/−^ mice on normal (A and C) or MCD diet (B and D), respectively, are shown. Arrowheads indicate isolated Kupffer cells whereas arrows show macrophage clusters. The abundance of isolated cells and clusters was estimated in 12 and 11 livers for WT mice, and 10 and 10 for IL-6^−/−^ mice fed a normal or MCD diet, respectively (** p<0.01, *** p<0.001 when comparing different diets for the same genotype).

Finally, to check the changes in F4/80 mRNA, the activation of Kupffer cells was studied histologically by examining F4/80-positive cells in the liver samples. In the animals fed a normal diet, Kupffer cells remained isolated, being only present in sinusoids (Fig. 7A and C). MCD diet feeding caused recruitment of Kupffer cells into clusters (Fig. 7B and D). While macrophage clusters seemed to be less abundant in IL-6^−/−^ mice, the difference did not reach statistical significance (p = 0.09).

## Discussion

The role of inflammatory cytokines in the development of NASH has not yet been fully elucidated. Despite recent studies on different rodent models, the contribution of TNFα to the pathogenesis of NASH remains unclear. Indeed, studies on mice deficient for TNFα or its receptors using different conditions to induce liver steatosis have not led to unequivocal conclusions. The emerging idea is that TNFα is not a primary mediator of NASH but influences its development [9], [10], [12]. As to IL-6, its contribution to NASH has remained almost totally elusive. Using a well-established nutritional model of NASH induction [13]–[15], the present study demonstrates that IL-6 certainly participates to the NASH-associated inflammation but is dispensable for the appearance of the disease. Whereas liver inflammation was markedly reduced in mice deficient for IL-6, as revealed both histologically and by analysing gene expression, liver injury and changes in lipid composition and peroxidation still occurred in mutant mice.

This is the first study to shed light on the role of IL-6 in a dietary model of NASH. In this model, IL-6 does not constitute the critical driving force for NASH but appears to modulate the inflammatory response rather than the initiation of the steatosis itself. In mice, IL-6 deficiency has been reported to induce a mature-onset obesity that appears after 6 months of life [42]. In the latter study, an increase in blood triglyceride levels was found that occurred only in females. The effect of IL-6 was supposed to be central. Of note, such an effect was not observed in the present study since we used younger males that lost weight on the MCD diet and that were slightly lighter than controls. Moreover, triglyceride levels equally decreased in both genotypes fed the MCD diet. These data strongly suggest that the alterations in lipid metabolism previously described in aged IL6-deficient females do not interfere with the present findings and interpretation.

IL-6 expression is known to be stimulated upon TNFα-mediated activation of NF-κB, a transcription factor believed to play a central role in NASH [40]. Not only has IL-6 been linked to insulin resistance [43] but also IL-6 level is increased in serum of patients with NASH [44], NAFLD [45] or alcoholic hepatitis [46]. The role of IL-6 in hepatic dysfunction in general, and insulin sensitivity and NASH in particular, remains, however, unclear [47]–[49]. Indeed, administration of IL-6 to IL-6^−/−^ mice was shown to prevent the massive liver necrosis after partial hepatectomy, suggesting an important role of this cytokine in hepatocyte regeneration [50]. Moreover, IL-6-deficient mice were highly susceptible to sepsis-induced steatosis and hepatocellular injury [51]. More recently, treatment of mice with IL-6 ameliorated steatosis in different models of fatty liver, including *ob/ob* mice and ethanol-fed mice [52]–[54]. Whether IL-6 plays a protective or injurious role on liver may depend on the duration of exposure to stress [30]. Furthermore, the cytokine action may be influenced by the local milieu [55] and, as examplified by the case of IL-6 on insulin sensitivity, the duality of IL-6 functions is likely tissue-specific and physiological context-dependent [56].

Here we show that genetic deletion of IL-6 markedly attenuates hepatic inflammation in MCD diet-induced NASH. This effect was observed both histologically and by quantitation of hepatic expression of genes that regulate the inflammatory response and promote liver fibrosis. Histologically, upon hematoxylin/eosin staining, whereas the nature of inflammatory cells (i.e., neutrophils and lymphocytes; data not shown) was similar in control and IL-6-deficient mice, there was less lobular inflammation in mutant mice. This was concordant with F4/80 staining. The most prominent difference between WT and IL-6-deficient animals was a virtually complete abrogation in mutant mice of the diet-induced increase in the expression of MCP-1 and TGF-β, i.e., factors that recruit inflammatory cells and activate hepatic stellate cells, respectively. Along with the reduction in TGF-β expression, the induction of Foxp3 was suppressed in IL-6-deficient mice, suggesting that in this disease model IL-6 modulates differentiation of Foxp3-positive T regulatory cells through TGF-β production. Whether Th17 cells, whose differentiation is also modulated by IL-6 [57], are involved in NASH would require further investigation. The possibility that SOCS (suppressor of cytokine signaling) proteins, known as critical targets of IL-6 in liver, serve as downstream mediators in this dietary model of NASH needs to be investigated. Also, the cellular origin of IL-6 would deserve careful investigation. To date, the cells that produce or react to IL-6 within the liver are not really known. Both Kupffer cells and hepatocytes are candidates. The IL-6 receptor is present on hepatocytes and some leukocytes but other cells can interact with a complex of IL-6 and its soluble receptor [58].

The present study further shows alterations in lipid homeostasis in NASH and provides novel insights into the role of these modifications to the initiation of associated inflammation. Regarding the changes in the liver content of PC and PE, which we observed and are expected because of the depletion of both choline and methionine [16], it was recently suggested that the relative depletion of PC or, perhaps more importantly, the decreased PC/PE ratio results in a loss of membrane integrity and influences liver damage [33]. Whether such a decrease in PC/PE ratio is the cause or consequence of progression to steatohepatitis was not established. Our study now indicates that the phospholipid changes precede, rather than follow, the inflammatory process since similar lipid alterations were seen in mice where inflammation is partly halted.

Whereas changes in the liver glycerophospholipid content have already been documented, this is the first study to report changes in sphingolipids, i.e., sphingomyelin and ceramide, in MCD diet-induced NASH. Irrespective of the cytokine defect, we found an elevation of hepatic levels of these two lipids. This is in accordance with a very recent lipidomic study that described a trend of increased hepatic content of sphingomyelin in patients with NASH [34]. The mechanisms and biological implications of these changes to the pathogenesis of NASH are still far from clear. It is possible that hepatocytes increase the synthesis of sphingomyelin, a membrane choline-containing phospholipid analogous to PC, to compensate for the loss of glycerophospholipids. Such synthesis of sphingomyelin probably occurs through increased *de novo* synthesis of ceramide, possibly because of enhanced incorporation of free fatty acids. Given the reported role of ceramide in hepatocellular death and, in general, as a bioactive lipid with pro-apoptotic properties [36], [59], it is tempting to speculate that the increased content of ceramide participates to NASH liver injury. Generation of this ceramide seems to be partially dependent on the production of inflammatory cytokines since the liver ceramide content of IL-6^−/−^ mice did not increase as much as in WT animals. Still for unclear reasons, the basal levels of both sphingomyelin and ceramide were higher in IL-6^−/−^ mice as compared to control animals; however, when normalized to total phospholipids, the basal ceramide levels were identical in both genotypes. Whether ceramide plays an instrumental role in the development of steatohepatitis remains to be investigated.

In conclusion, the present study demonstrates that IL-6 does contribute to the inflammatory process associated with the development of NASH. Because the dietary model of NASH we used does not induce obesity or insulin resistance, i.e. important features of the human condition, future studies using different models of NASH should shed further light on the role of IL-6 in the development of steatohepatitis. Of particular interest is the recent observation that IL-6 expression is increased in the liver of patients with NASH, is associated with elevation of circulating IL-6 levels, and correlates with disease severity [60]. Further attention should thus be paid to IL-6 as a potential pathogenic mechanism in NASH.

## References

[1] Ludwig J, Viggiano TR, McGill DB, Oh BJ (1980). Nonalcoholic steatohepatitis: Mayo Clinic experiences with a hitherto unnamed disease.. Mayo Clin Proc.

[2] Haque M, Sanyal AJ (2002). The metabolic abnormalities associated with non-alcoholic fatty liver disease.. Best Pract Res Clin Gastroenterol.

[3] Day CP, James OF (1998). Steatohepatitis: a tale of two “hits”?. Gastroenterology.

[4] Larter CZ, Farrell GC (2006). Insulin resistance, adiponectin, cytokines in NASH: Which is the best target to treat?. J Hepatol.

[5] Hotamisligil GS (1999). The role of TNFalpha and TNF receptors in obesity and insulin resistance.. J Intern Med.

[6] Yin M, Wheeler MD, Kono H, Bradford BU, Gallucci RM (1999). Essential role of tumor necrosis factor alpha in alcohol-induced liver injury in mice.. Gastroenterology.

[7] Crespo J, Cayon A, Fernandez-Gil P, Hernandez-Guerra M, Mayorga M (2001). Gene expression of tumor necrosis factor alpha and TNF-receptors, p55 and p75, in nonalcoholic steatohepatitis patients.. Hepatology.

[8] Bahcecioglu IH, Yalniz M, Ataseven H, Ilhan N, Ozercan IH (2005). Levels of serum hyaluronic acid, TNF-alpha and IL-8 in patients with nonalcoholic steatohepatitis.. Hepatogastroenterology.

[9] Memon RA, Grunfeld C, Feingold KR (2001). TNF-alpha is not the cause of fatty liver disease in obese diabetic mice.. Nat Med.

[10] Deng QG, She H, Cheng JH, French SW, Koop DR (2005). Steatohepatitis induced by intragastric overfeeding in mice.. Hepatology.

[11] Feldstein AE, Werneburg NW, Canbay A, Guicciardi ME, Bronk SF (2004). Free fatty acids promote hepatic lipotoxicity by stimulating TNF-alpha expression via a lysosomal pathway.. Hepatology.

[12] Tomita K, Tamiya G, Ando S, Oshumi K, Chiyo T (2006). Tumour necrosis factor alpha signalling through activation of Kupffer cells plays an essential role in liver fibrosis of non-alcoholic steatohepatitis in mice.. Gut.

[13] McCuskey RS, Ito Y, Robertson GR, McCuskey MK, Perry M (2004). Hepatic microvascular dysfunction during evolution of dietary steatohepatitis in mice.. Hepatology.

[14] Rinella ME, Green RM (2004). The methionine-choline deficient dietary model of steatohepatitis does not exhibit insulin resistance.. J Hepatol.

[15] Diehl AM (2005). Lessons from animal models of NASH.. Hepatol Res.

[16] Yao ZM, Vance DE (1988). The active synthesis of phosphatidylcholine is required for very low density lipoprotein secretion from rat hepatocytes.. J Biol Chem.

[17] Rinella ME, Elias MS, Smolak RR, Fu T, Borensztajn J (2008). Mechanisms of steatohepatitis in mice fed a lipogenic methionine choline deficient (MCD) diet.. J Lipid Res.

[18] Kopf M, Baumann H, Freer G, Freudenberg M, Lamers M (1994). Impaired immune and acute-phase responses in interleukin-6-deficient mice.. Nature.

[19] Brunt EM, Janney CG, Di Bisceglie AM, Neuschwander-Tetri BA, Bacon BR (1999). Nonalcoholic steatohepatitis: a proposal for grading and staging the histological lesions.. Am J Gastroenterol.

[20] Kleiner DE, Brunt EM, Van Natta M, Behling C, Contos MJ (2005). Design and validation of a histological scoring system for nonalcoholic fatty liver disease.. Hepatology.

[21] Bradford MM (1976). A rapid and sensitive method for the quantitation of microgram quantities of protein utilizing the principle of protein-dye binding.. Anal Biochem.

[22] Folch J, Lees M, Sloane Stanley GH (1957). A simple method for the isolation and purification of total lipides from animal tissues.. J Biol Chem.

[23] Ames BN (1966). Assay of inorganic phosphate, total phosphate and phosphatases.. Meth Enzymol.

[24] Bielawska A, Perry DK, Hannun YA (2001). Determination of ceramides and diglycerides by the diglyceride kinase assay.. Anal Biochem.

[25] George J, Pera N, Phung N, Leclercq I, Yun Hou J (2003). Lipid peroxidation, stellate cell activation and hepatic fibrogenesis in a rat model of chronic steatohepatitis.. J Hepatol.

[26] Cuvillier O, Edsall L, Spiegel S (2000). Involvement of sphingosine in mitochondria-dependent Fas-induced apoptosis of type II Jurkat T cells.. J Biol Chem.

[27] Natori S, Rust C, Stadheim LM, Srinivasan A, Burgart LJ (2001). Hepatocyte apoptosis is a pathologic feature of human alcoholic hepatitis.. J Hepatol.

[28] Feldstein AE, Canbay A, Angulo P, Taniai M, Burgart LJ (2003). Hepatocyte apoptosis and fas expression are prominent features of human nonalcoholic steatohepatitis.. Gastroenterology.

[29] Ribeiro PS, Cortez-Pinto H, Sola S, Castro RE, Ramalho RM (2004). Hepatocyte apoptosis, expression of death receptors, and activation of NF-kappaB in the liver of nonalcoholic and alcoholic steatohepatitis patients.. Am J Gastroenterol.

[30] Jin X, Zimmers TA, Perez EA, Pierce RH, Zhang Z (2006). Paradoxical effects of short- and long-term interleukin-6 exposure on liver injury and repair.. Hepatology.

[31] Kirsch R, Clarkson V, Verdonk RC, Marais AD, Shepard EG (2006). Rodent nutritional model of steatohepatitis: effects of endotoxin (lipopolysaccharide) and tumor necrosis factor alpha deficiency.. J Gastroenterol Hepatol.

[32] Baumgardner JN, Shankar K, Hennings L, Badger TM, Ronis MJ (2007). A New Model For Non-Alcoholic Steatohepatitis in the Rat Utilizing Total Enteral Nutrition to Overfeed a High Polyunsaturated Fat Diet.. Am J Physiol Gastrointest Liver Physiol.

[33] Li Z, Agellon LB, Allen TM, Umeda M, Jewell L (2006). The ratio of phosphatidylcholine to phosphatidylethanolamine influences membrane integrity and steatohepatitis.. Cell Metab.

[34] Puri P, Baillie RA, Wiest MM, Mirshahi F, Choudhury J (2007). A lipidomic analysis of nonalcoholic fatty liver disease.. Hepatology.

[35] Holland WL, Brozinick JT, Wang LP, Hawkins ED, Sargent KM (2007). Inhibition of ceramide synthesis ameliorates glucocorticoid-, saturated-fat-, and obesity-induced insulin resistance.. Cell Metab.

[36] Mari M, Fernandez-Checa JC (2007). Sphingolipid signalling and liver diseases.. Liver Int.

[37] Malagarie-Cazenave S, Andrieu-Abadie N, Ségui B, Gouaz V, Tardy C (2002). Sphingolipid signalling: molecular basis and role in TNF–induced cell death.. Expert Rev Mol Med 2002..

[38] Malagarie-Cazenave S, Ségui B, Leveque S, Garcia V, Carpentier S (2004). Role of FAN in tumor necrosis factor-alpha and lipopolysaccharide-induced interleukin-6 secretion and lethality in D-galactosamine-sensitized mice.. J Biol Chem.

[39] Leclercq IA, Farrell GC, Field J, Bell DR, Gonzalez FJ (2000). CYP2E1 and CYP4A as microsomal catalysts of lipid peroxides in murine nonalcoholic steatohepatitis.. J Clin Invest.

[40] Dela Pena A, Leclercq I, Field J, George J, Jones B (2005). NF-kappaB activation, rather than TNF, mediates hepatic inflammation in a murine dietary model of steatohepatitis.. Gastroenterology.

[41] Gyamfi MA, Damjanov I, French S, Wan YJ (2008). The pathogenesis of ethanol versus methionine and choline deficient diet-induced liver injury.. Biochem Pharmacol.

[42] Wallenius V, Wallenius K, Ahren B, Rudling M, Carlsten H (2002). Interleukin-6-deficient mice develop mature-onset obesity.. Nat Med.

[43] Senn JJ, Klover PJ, Nowak IA, Mooney RA (2002). Interleukin-6 induces cellular insulin resistance in hepatocytes.. Diabetes.

[44] Abiru S, Migita K, Maeda Y, Daikoku M, Ito M (2006). Serum cytokine and soluble cytokine receptor levels in patients with non-alcoholic steatohepatitis.. Liver Int.

[45] Haukeland JW, Damas JK, Konopski Z, Loberg EM, Haaland T (2006). Systemic inflammation in nonalcoholic fatty liver disease is characterized by elevated levels of CCL2.. J Hepatol.

[46] Hill DB, Marsano L, Cohen D, Allen J, Shedlofsky S (1992). Increased plasma interleukin-6 concentrations in alcoholic hepatitis.. J Lab Clin Med.

[47] Pedersen BK, Fischer CP (2007). Physiological roles of muscle-derived interleukin-6 in response to exercise.. Curr Opin Clin Nutr Metab Care.

[48] Mooney RA (2007). Counterpoint: Interleukin-6 does not have a beneficial role in insulin sensitivity and glucose homeostasis.. J Appl Physiol.

[49] Pedersen BK, Febbraio MA (2007). Point: Interleukin-6 does have a beneficial role in insulin sensitivity and glucose homeostasis.. J Appl Physiol.

[50] Cressman DE, Greenbaum LE, DeAngelis RA, Ciliberto G, Furth EE (1996). Liver failure and defective hepatocyte regeneration in interleukin-6-deficient mice.. Science.

[51] Deutschman CS, Cereda M, Ochroch EA, Raj NR (2006). Sepsis-induced cholestasis, steatosis, hepatocellular injury, and impaired hepatocellular regeneration are enhanced in interleukin-6 −/− mice.. Crit Care Med.

[52] Hong F, Radaeva S, Pan HN, Tian Z, Veech R (2004). Interleukin 6 alleviates hepatic steatosis and ischemia/reperfusion injury in mice with fatty liver disease.. Hepatology.

[53] Hong F, Kim WH, Tian Z, Jaruga, Ishac E (2002). Elevated interleukin-6 during ethanol consumption acts as a potential endogenous protective cytokine against ethanol-induced apoptosis in the liver: involvement of induction of Bcl-2 and Bcl-x(L) proteins.. Oncogene.

[54] El-Assal O, Hong F, Kim WH, Radaeva S, Gao B (2004). IL-6-deficient mice are susceptible to ethanol-induced hepatic steatosis: IL-6 protects against ethanol-induced oxidative stress and mitochondrial permeability transition in the liver.. Cell Mol Immunol.

[55] McGettrick HM, Smith E, Filer A, Kissane S, Salmon M (2009). Fibroblasts from different sites may promote or inhibit recruitment of flowing lymphocytes by endothelial cells.. Eur J Immunol.

[56] Kim JH, Bachmann RA, Chen J (2009). Interleukin-6 and insulin resistance.. Vitam Horm.

[57] Korn T, Betteli E, Oukka M, Kuchroo VK (2009). IL-17 and Th17 Cells.. Annu Rev Immunol.

[58] Scheller J, Rose-John S (2006). Interleukin-6 and its receptor: from bench to bedside.. Med Microbiol Immunol.

[59] Fernandez-Checa JC, Colell A, Mari M, Garcia-Ruiz C (2005). Ceramide, tumor necrosis factor and alcohol-induced liver disease.. Alcohol Clin Exp Res.

[60] Wieckowska A, Papouchado BG, Li Z, Lopez R, Zein NN (2008). Increased hepatic and circulating interleukin-6 levels in human nonalcoholic steatohepatitis.. Am J Gastroenterol.

